# Accuracy of Age Estimation Using Three Dental Age Estimation Methods in a Young, Large, and Multiethnic Patient Sample

**DOI:** 10.3390/dj11120288

**Published:** 2023-12-13

**Authors:** Claire Willmann, Gabriel Fernandez De Grado, Céline Kolb, Jean-Sébastien Raul, Anne-Marie Musset, Catherine-Isabelle Gros, Damien Offner

**Affiliations:** 1 Faculté de Chirurgie Dentaire, Université de Strasbourg, 8 Rue Sainte Elisabeth, 67000 Strasbourg, France; fernandezdegrado@unistra.fr (G.F.D.G.); celine.kolb@unistra.fr (C.K.); musset@unistra.fr (A.-M.M.); catherine-isabelle.gros@chru-strasbourg.fr (C.-I.G.); damien.offner@chru-strasbourg.fr (D.O.); 2 Pôle de Médecine et Chirurgie Bucco-Dentaires, Hôpitaux Universitaires de Strasbourg (HUS), 67000 Strasbourg, France; 3 INSERM (French National Institute of Health and Medical Research), UMR 1260, Regenerative Nanomedicine (RNM), FMTS, CRBS, 1 Rue Emile Boeckel, 67084 Strasbourg, France; 4 ICube—UMR 7357 Institut de Médecine Légale, 11 rue Humann, 67085 Strasbourg, France; js.raul@unistra.fr

**Keywords:** age estimation, dental age, forensic odontology, orthopantomogram

## Abstract

European countries have become host countries for migrants and unaccompanied minors. However, many migrants arrive without identity documents. Many methods exist to estimate age; among them, several methods using dental age have been proposed. Our objective was to evaluate the accuracy of biological age determination in a multiethnic sample using dental age estimated using three methods: Nolla, Demirjian, and the London Atlas. Orthopantomograms collected for 324 patients of various ethnicities aged from 4 to 20 years old were included. Then, for each orthopantomogram, a blind trained examiner used the three methods of age estimation. For each method, the estimated mean age was greater than the real mean age (*p* < 0.0001). The accuracy after 18 years old with a 1-year margin was under 50%. Demirjian’s method gave a less accurate estimated age than Nolla’s method (*p* < 0.0001) or the London Atlas (*p* < 0.001). The most accurate methods were those of Nolla and the London Atlas, with average absolute deviations of 1.3 and 1.2 years, respectively. Demirjian’s method was much less accurate, with a deviation of around 2 years. The evaluated methods are unable to provide reliable information to determine if an individual is a minor.

## 1. Introduction

The United Nations Convention on the Rights of the Child defines an unaccompanied minor as a child or adolescent under the age of 18 who is “separated from both parents and other relatives and is not being cared for by any other adult who, by law or custom, is responsible for doing so” [1,2,3]. On a worldwide scale, the number of migrant children was 33 million in 2019 [4]. Since 1990, the number of international child migrants has grown along with the global population, with the share of migrants among the world’s children remaining stable [5]. According to UNICEF, children migrate to escape violence, armed conflict, persecution, the ravages of climate change and natural disasters, poverty, and inequality. Some of them are orphans in their countries or want to join relatives who have already emigrated. Their reasons for migrating may evolve and overlap. Some families also send their children alone because they know that unaccompanied minors have a special status and are more likely to be allowed to stay in the host country [5]. Migration routes are extremely dangerous, and legal means of immigration are often limited, especially for unaccompanied minors. They are more vulnerable than adults and can easily find themselves in the hands of smugglers and become victims of human trafficking, abuse, and persecution [1,5]. Within Europe, France has become a host country for migrants and unaccompanied minors [3].

In 2020, 13,550 migrants in Europe, including 650 in France, applied for asylum as unaccompanied minors [1,6]. This country provides special status and protection for these unaccompanied minors in accordance with the International Convention on the Rights of the Child, signed on 20 November 1989 and applied since 6 September 1990 [7].

Many migrants arrive without identity documents or with documents for which the validity cannot be verified. It is a delicate but necessary procedure to determine whether young migrants are legally entitled to a special status because of their minority. Similarly, for the perpetrators or victims of crimes or delinquencies, the legal consequences will be different depending on whether they are minors.

According to the Council of Europe recommendations, age determination procedures must be carried out in accordance with the fundamental rights of the child, respecting gender, dignity, and cultural differences. Physical examinations and other forms of medical examinations, such as X-rays of the jaw or carpal bones, should be measures of last resort. Other alternative comprehensive methods, such as collecting and using documentary evidence and conducting an age assessment interview with the concerned person, should be preferred [8].

In France, there is no consensus regarding the method of age determination. French law allows bone X-ray examinations for age determination purposes in the absence of valid identity documents and when the alleged age is not credible. These examinations can only be carried out after a decision is made by the judicial authority and after obtaining the consent of the concerned person. Their conclusions, which must specify the margin of error, cannot in themselves determine whether the concerned person is a minor and must be associated with other scientific and administrative sources [9].

Dental age estimation is used in pediatric and orthodontic dentistry. It is also used to estimate the ages of archaeological subjects in past populations and in forensic dentistry [10,11,12].

There are many evaluation methods for dental age assessment. It is widely accepted that an analysis of dental development based on calcification is a better age indicator than eruption [13,14]. Indeed, eruption can be influenced by many factors, such as the premature loss of temporary teeth, a lack of space on the dental arch, dental caries, or malnutrition. In addition, tooth eruption is a short-term phenomenon, whereas maturation and calcification are continuous processes over many years. Thus, the study of dental development allows the assessment of dental maturity over periods when no eruption takes place [13,15]. Dental calcification is a widely used method of age estimation, as it is less influenced by environmental variations (nutrition, heredity, metabolism, etc.) than other methods based on sexual or skeletal development.

Several methods for determining dental age via the calcification on a panoramic radiograph have been proposed, including Nolla’s method [16], Demirjian’s method [15], and the London Atlas [17].

Nolla’s method uses 10 stages of tooth calcification on permanent teeth. Each maxillary or mandibular tooth is assigned a value from 1 to 10 [16]. If a tooth is between two stages, a value of 0.2, 0.5, or 0.7 is added. For example, if a tooth is between stages 6 and 7 but is closer to stage 6, it is scored 6.2. If it is closer to stage 7, it is scored 6.7, and if it is an equal distance from 6 and 7, it is scored 6.5. The tooth development on the left side of the jaw is almost identical to that on the right side; only one value is retained per pair of teeth. The values are summed for the mandible, the maxillary, or both and compared to Nolla’s tables according to sex, accounting for the presence or absence of the third molars, to determine the estimated age of each individual.

Demirjian’s method uses a similar approach based on eight stages of calcification of the seven mandibular left-side teeth. These stages are then converted into numerical values, and standard tables assign each total maturity score to a given dental age. This method can only be used up to 16 years old and excludes wisdom teeth [15]. After this age, Olze’s method based on the mineralization status of the third molars can complete the analysis [18].

A third method, the London Atlas of Human Tooth Development and Eruption, is based on a visual comparison of tooth development and alveolar eruption on the right side of the jaw with standard figures (available at https://www.qmul.ac.uk/dentistry/atlas, accessed on 1 November 2022). It covers the period between 28 weeks in utero and 23 years old and makes no distinctions between males and females [17].

Most of these methods were developed in homogeneous Caucasian populations and may not be appropriate to use in every situation, especially when estimating the ages of migrants with extremely diverse origins.

In this context, our objective was to evaluate the accuracy of biological age determination in a multiethnic sample using dental age estimated via three methods: Nolla, Demirjian, and the London Atlas.

## 2. Materials and Methods

Patients who attended the dental clinic of Strasbourg’s public university hospital (Pôle de Médecine et Chirurgie Bucco-dentaires des Hôpitaux Universitaires de Strasbourg) between January and September 2018 and for whom an orthopantomogram (OPT, i.e., panoramic radiography) was already available were considered for inclusion.

The included population from Strasbourg is multiethnic, including French individuals (20% of them from another ethnicity) [19] as well as migrants with legal or illegal status from many other countries, especially from Eastern Europe, Africa, and the Middle East. It was not possible to record the details of each individual’s ethnicity, but it is likely that immigrants are overrepresented, as the public hospital is the main place of appeal for migrants needing dental treatment. Ages were obtained from identity documents.

All OPTs were obtained using the same X-ray generator (Promax Digital Panoramic, Planmeca, Helsinki, Finland). The radiological constants used were 62 to 66 kV, 5 to 8 mA, and 13 to 15 s (subjects from 5 to 10 years old); 64 to 68 kV, 7 to 14 mA, and 15 s (subjects from 11 to 15 years old); and 66 to 70 kV, 7 to 11 mA, and 15 s (subjects from 15 to 20 years old).

The inclusion criteria for the panoramic radiographies were as follows:

− Both mandibular branches had the same width.
− The occlusal plane was horizontal or slightly concave.
− Non-blurry, clear image of the tooth apexes.
− No sign of periapical infections or endodontic treatments on any tooth.

An experienced senior employee from the dental radiography unit examined all OPTs and selected those meeting the inclusion criteria. The images were then anonymized, and information about the patient’s age was removed. Information about sex was preserved to allow the correct use of each method. Then, for each OPT, a blind trained examiner consecutively used the three methods of age estimation described in the introduction: Nolla, Demirjian (and Olze), and the London Atlas of Human Tooth Development and Eruption.

Nolla’s method was used first by assessing the development and calcification of the left mandibular teeth to determine the age of the subject.

For Demirjian’s method, each of the seven mandibular teeth on the left side were assigned a corresponding stage of maturation, from A to H, which was then converted into a numerical value. If the score was exactly between two scores, the average age value was used. Olze’s method [18] was then applied for individuals whose ages estimated with the Demirjian method were greater than 16. The development of tooth 38 was first assessed according to Demirjian’s stages. Then, using a table given by Olze, the dental age corresponding to its stage was determined. If tooth 38 was missing, tooth 48 was analyzed.

Finally, OPTs were compared to the dental diagrams in the London Atlas; each image was assigned an age according to the most similar figure, with a precision of 0.5 to 1 year. If a tooth was between two stages of development, the average value was used.

Statistical analyses were performed using Excel (Microsoft, Redmont, WA, USA) and R (R Core Team (2018)).

**Figure 1:**
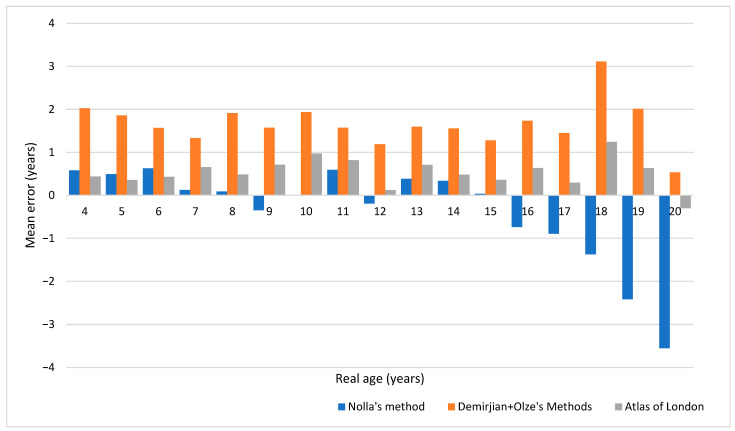
Means of errors for each method and each age category.

For each method, the estimated age was compared with the real age using Student’s *t*-test for paired samples after checking for its applicability. For each individual and each method, the absolute value of the difference (delta/Δ) between the real age (with a one-month precision) and the estimated age was calculated and used to determine each method’s accuracy (Table 1). The non-absolute difference was used to illustrate tendencies toward over- or underestimation (Figure 1 and Table 1).

## 3. Results

After selection, 324 OPTs from 324 patients (156 males and 168 females) aged from 4 to 20 years old were included (Table 1).

**Figure 2:**
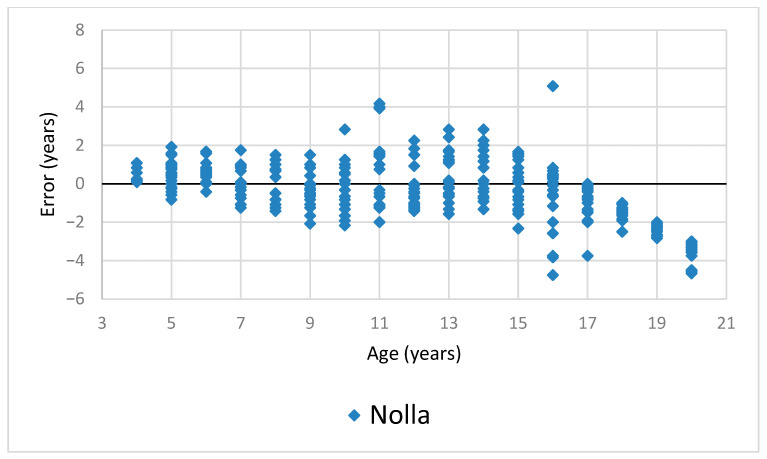

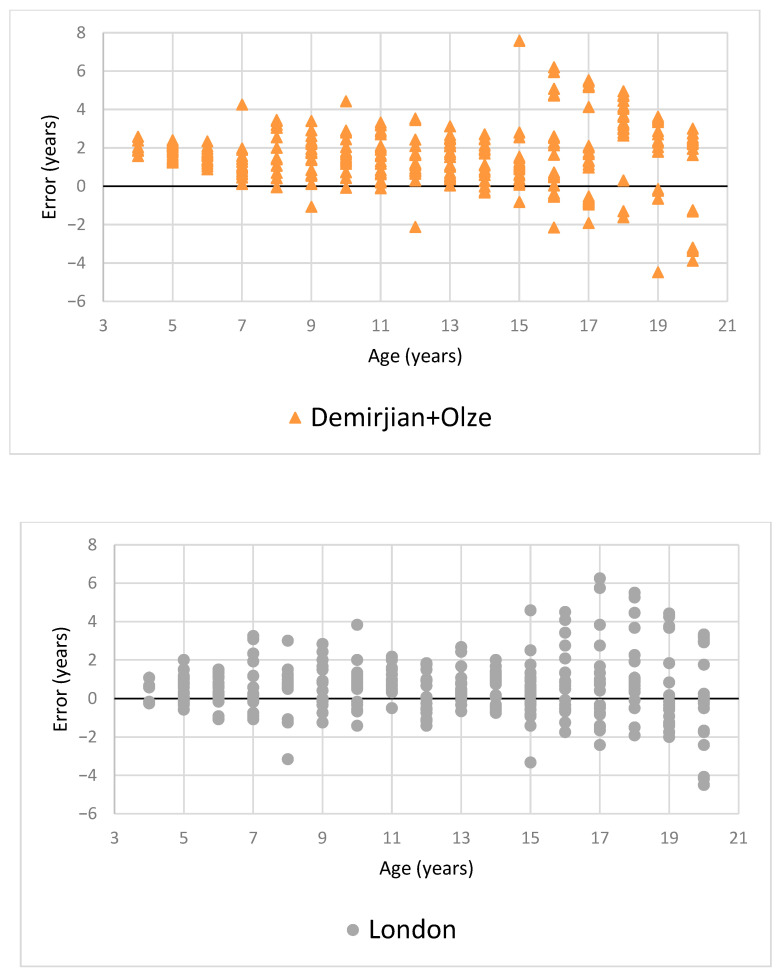
Scatter plots of estimations for each individual for each method by age.

The means of the errors for each age (age categories rounded to the lowest) and method are presented in Figure 1 and Table 1. A full scatter plot of the estimations for each method is shown in Figure 2.

Overall, for each method, the mean estimated age was greater than the real age (*p* < 0.0001). Demirjian’s method resulted in a mean of absolute error between the estimated and real ages of 1.93 years, which was much greater than the values obtained using Nolla’s method (1.25 years; *p* < 0.0001) or the London Atlas (1.16 years; *p* < 0.001). The difference observed between Nolla’s method and the London Atlas was not significant (*p* = 0.39).

The proportions of correct estimations for each age with margins of 1 and 2 years are presented in Table 2.

Regardless of the age, no method managed to guarantee a correct estimation with a 1-year margin for 100% of the subjects.

After 18 years old, all methods tended to lose precision, especially Nolla’s method, which gradually underestimated real age after 16 years old. The London Atlas performed slightly better than the other methods after 18 years old.

Sex made no significant difference in either of the analyses, and no tendency for better or worse estimation was observed for either sex.

## 4. Discussion

The objective of this study was to evaluate the accuracy of biological age determination in a multiethnic sample using dental age estimated via three methods: Nolla, Demirjian, and the London Atlas.

The results show that no studied method ensured a satisfactory estimation of age, especially after 16 years old, the age at which the accuracy with a 1-year margin was reduced for all studied methods. This is especially problematic since the age of majority in almost all European countries is 18 years old. Thus, the three methods become less precise once all teeth have completed their development. According to our analysis, the most accurate methods are those of Nolla and the London Atlas, which have average absolute deviations of 1.3 and 1.2 years, respectively. The other method, combining Demirjian’s and Olze’s methods, is much less accurate, with a deviation of around 2 years.

The performances we observed were even worse than those reported in other studies, especially using Demirjian’s method, for which previous studies reported an overestimation of one year [15,20,21]. Most methods were established and tested in homogenous Caucasian populations: French Canadians for Demirjian’s method, Whites and Bangladeshis for the London Atlas, and likely Caucasian Americans for Nolla’s method. Poorer precision was expected when working with a multiethnic sample, an expectation confirmed by our results. It is important to note that for Demirjian’s method, the original tables from 1973 based on French Canadians were used, which seemed adequate for a French population. Other tables were proposed over time. In particular, a table by Willems based on Belgian children could also have been expected to apply to our sample in France [22]. A few observations with Willems’ tables on a subsample of our population (results not published) showed slightly better estimations than with the original tables, but the results were still less accurate than using other methods.

These results are in accordance with those of different authors who compared the methods of Nolla and Demirjian and concluded that the Nolla method is more accurate [23,24,25]. This was further mentioned by Tomas et al., who highlighted that, in adults, neither method is able to estimate age accurately [23].

In terms of speed of execution, the easiest and quickest method to use was the London Atlas, followed by the Nolla and Demirjian/Olze methods.

Cameriere et al. developed different methods to evaluate dental age in recent years. One of them, which uses Bayesian calibration, initially reported better results but was only tested on children aged from 5 to 17 years old [26]. Further studies evaluating this method mostly focused on children under 15 years old, according to a 2023 meta-analysis [27]. Just like the methods we evaluated, Cameriere’s method shows a loss of accuracy after 14 years old [27].

However, another method developed by Cameriere using a third-molar maturity index seems to show much better accuracy and could be used to determine if an individual is over 18 years old with 99.9% probability in some situations when combined with other physical and radiological examinations [28]. This method has been adapted to many ethnicities [29,30].

Modern dental age estimation may achieve better accuracy using statistical tools such as regression and Bayesian calibration or even using artificial-intelligence-based approaches [31]. These methods require strict evaluations of their accuracy, especially for children and young adults aged around 18 years old, if their goal is to help evaluate an individual’s minority according to the United Nations Convention on the Rights of the Child.

## 5. Conclusions

None of the methods that we compared could determine biological age with adequate accuracy to remove any legal doubt, especially for individuals close to the age of majority, even with a 2-year margin. However, the Nolla and London Atlas methods should be preferred, as they have a higher degree of accuracy than the other method evaluated here.

Although the accuracy of these dental age evaluation methods decreases with increases in real age, they are complementary to other existing age determination techniques. They could be coupled with bone age methods, oral interviews, and the examination of legal documents in order to better refine the assessment of the real ages of patients.

## Figures and Tables

**Table 1:** Description of the sample: mean error and mean of absolute errors (in years) for each method at each age.

Age Categories (Rounded to Lowest)	Number of Subjects (% of Total Sample)	Nolla		Demirjian + Olze		London Atlas	
		Mean Error	Mean of Absolute Errors	Mean Error	Mean of Absolute Errors	Mean Error	Mean of Absolute Errors
4	7 (2.16%)	0.58	0.58	2.02	2.02	0.44	0.56
5	26 (8.02%)	0.49	0.72	1.86	1.86	0.36	0.49
6	18 (5.56%)	0.63	0.67	1.57	1.57	0.43	0.67
7	18 (5.56%)	0.13	0.65	1.33	1.33	0.65	1.00
8	14 (4.32%)	0.09	0.89	1.91	1.92	0.48	1.27
9	16 (4.94%)	−0.35	0.82	1.57	1.71	0.72	1.05
10	20 (6.17%)	0.00	1.23	1.94	1.94	0.97	1.29
11	20 (6.17%)	0.59	1.61	1.57	1.58	0.82	0.87
12	19 (5.86%)	−0.19	0.88	1.19	1.41	0.12	0.89
13	20 (6.17%)	0.38	1.02	1.59	1.59	0.71	0.82
14	21 (6.48%)	0.34	1.09	1.56	1.62	0.48	0.85
15	20 (6.17%)	0.03	1.03	1.28	1.36	0.36	1.14
16	20 (6.17%)	−0.74	1.23	1.73	2.09	0.63	1.27
17	24 (7.41%)	−0.89	0.89	1.45	2.16	0.30	1.75
18	21 (6.48%)	−1.37	1.37	3.11	3.39	1.25	1.67
19	20 (6.17%)	−2.42	2.42	2.02	2.57	0.63	1.70
20	20 (6.17%)	−3.55	3.55	0.54	2.49	−0.30	2.08
**Total**	**324 (100.00%)**	**−0.42**	**1.25**	**1.65**	**1.93**	**0.53**	**1.16**

**Table 2:** Rates of correct estimations with 1-year and 2-year margins. Rates of correct estimations of 100% are highlighted in dark green. Rates of correct estimations over 50% are highlighted in light green.

	Rates of Correct Estimations with 1-Year and 2-Year Margins					
Age	Method					
	Nolla	Nolla	Demirjian + Olze		London	London
	1 Year	2 Years	1 Year	2 Years	1 Year	2 Years
4	71.43%	100.00%	0.00%	57.14%	85.71%	100.00%
5	73.08%	100.00%	0.00%	50.00%	76.92%	96.15%
6	83.33%	100.00%	5.56%	88.89%	66.67%	100.00%
7	77.78%	100.00%	33.33%	94.44%	61.11%	83.33%
8	50.00%	100.00%	21.43%	50.00%	35.71%	85.71%
9	62.50%	93.75%	25.00%	62.50%	56.25%	81.25%
10	52.63%	89.47%	15.79%	68.42%	47.37%	84.21%
11	20.00%	75.00%	35.00%	65.00%	70.00%	90.00%
12	52.63%	94.74%	47.37%	73.68%	57.89%	100.00%
13	45.00%	90.00%	30.00%	55.00%	75.00%	90.00%
14	52.38%	85.71%	42.86%	66.67%	61.90%	95.24%
15	45.00%	95.00%	50.00%	85.00%	65.00%	85.00%
16	66.67%	71.43%	42.86%	47.62%	57.14%	76.19%
17	62.50%	91.67%	41.67%	66.67%	37.50%	75.00%
18	0.00%	95.24%	4.76%	14.29%	47.62%	76.19%
19	0.00%	0.00%	15.00%	20.00%	45.00%	70.00%
20	0.00%	0.00%	0.00%	25.00%	35.00%	50.00%
**Total**	**46.91%**	**80.25%**	**25.00%**	**57.72%**	**57.10%**	**83.95%**

## Data Availability

Data are available upon request. Contact the main author.
